# Complete Upper Body Bar Enhances Strength Training During Bench Press

**DOI:** 10.3390/muscles4010007

**Published:** 2025-03-12

**Authors:** He Wang, Hannah Bradshaw, Ben VonGunten, John Andamasaris, Emma Burns, Caroline Ashton, Clark Dickin

**Affiliations:** Biomechanics Laboratory (HP 311B), Health and Physical Activity Building, School of Kinesiology, Ball State University, Muncie, IN 47306, USA; hannah.bradshaw@bsu.edu (H.B.); benjamin.vongunten@bsu.edu (B.V.); jandamasaris@bsu.edu (J.A.); emma.burns@bsu.edu (E.B.); caroline.ashton@bsu.edu (C.A.); dcdickin@bsu.edu (C.D.)

**Keywords:** bench press, EMG, pronator teres, supinator

## Abstract

Barbell (BB) and dumbbell (DB) devices are commonly used during a bench press to develop the muscles of the chest, shoulders, and upper arms. Recently, a complete upper body bar (CUBB) was designed to train the muscles of the forearm by allowing for pronation and supination while providing the same traditional training for the rest of the upper body. The purpose of this study was to investigate the effectiveness of the CUBB relating to the EMG activity of the forearm during a bench press. Methods: A total of 21 healthy college-aged men volunteered for this study. EMG sensors were placed on the anterior deltoid (AD), pectoralis major (PEC), triceps brachii (TRI), pronator teres (PRO), and supinator (SUP). The participants went through a bench press test in a series of three different randomized conditions: the DB, the BB, and the CUBB. Resistance was set at 30% of body weight. A repeated-measures ANOVA was used to analyze the normalized EMG data (alpha = 0.05). Results: For the forearm muscles, the CUBB exhibited 41% and 37% higher PRO activation than the DB and BB, respectively. In addition, the CUBB exhibited 67% and 30% more SUP activation than the BB and DB, respectively. For the shoulder and chest muscles (AD and PEC), no significant differences were found among the three conditions. Conclusions: Bench pressing with a CUBB can engage more upper body muscles and offer individuals additional training benefits.

## 1. Introduction

Upper body strength is essential for people to carry out everyday functionalities. For example, holding a basket or pushing a shopping cart at a grocery store requires some level of upper body muscle strength. Adequate upper body muscle strength could provide individuals with the necessary ability to maintain good posture [1]. Poor body postures such as kyphosis or lordosis can be self-corrected with improved upper body strength [2]. Upper body strength training also benefits individuals’ overall health, including bone health and muscle mass [3,4]. With a good reserve of large muscle forces and improved bone quality, an individual could easily alleviate the risk of fall-related injuries. For the athletic population, the demand for stronger upper body strength is even more prominent. Athletes engaging in striking or throwing events need a well-built upper body structure to deliver satisfactory performance. The upper body joints ranging from the proximal joints such as the shoulder to the distal joints such as the forearm and wrist must be conditioned to be able to work together to forcefully execute athletic movements (e.g., shot putting, baseball pitching, handball throwing), which utilize the upper body’s kinetic chain for efficient energy transfer from the proximal to the distal joints [5,6,7].

The bench press is an essential part of an upper body resistance training program. It is easy to implement. The training only requires some simple equipment such as a bench and a weighted bar bell or a pair of dumbbells to begin. The training effect is predictable and significant. The shoulder flexors and adductors (e.g., pectoralis major, anterior deltoid) as well as the elbow extensors (e.g., triceps brachii) are the muscles targeted by a bench press exercise [8,9]. The resistance comes from the weights of the devices applied in the vertical direction aligned with gravity. Adding weights to the devices is the means of increasing the resistance and promoting the muscles’ engagement during the exercise. Due to the above-mentioned benefits, the bench press is a widely used upper body resistance training protocol. Indeed, recreational and professional athletes consider the bench press as the most popular form of strength training for the upper body [10,11].

While it is understood that the intensity level of muscle activity corresponds to the level of resistance, limited research has been conducted to understand the effects of variations in the forms of the bench press on muscle engagement [9,10,11,12,13,14,15]. Factors including the grip distance, stability of the bench surface, sources of the resistance, and inclination of the bench were the foci of these studies [9,10,11,12,13,14,15]. The intergrip distance during a bench press has an effect on the engagement of the upper body muscles [12,13]. Studies investigating the relationship between the intergrip distance and muscle activation profiles showed that as the intergrip distance reduces, the activation level of the triceps brachii increases, while the pectoralis major reduces its activity [12,13]. Also, the stability of the bench surface could influence the outcomes of a bench press exercise [10]. It was reported that a stable bench helps promote higher strength output with greater activities in the pectoralis major and triceps brachii than other unstable surfaces including a balance cushion and a Swiss ball [10]. In addition, free-weight bench presses were found to activate more muscles than machine-assisted versions [9,14]. Specifically, a free-weight bench press promotes greater engagement in the medial and anterior deltoid than a machine bench press [14]. A free-weight press also activates the medial deltoid more than the Smith machine press [9]. Finally, alteration in the bench inclination angle could help address different upper body muscle groups [15]. A flat bench setting produces similar activities between the pectoralis major and the anterior deltoid. When the bench inclination becomes 30°, the upper portion of the pectoris major sees an increase in activation. With a 45° inclination, bench press promotes the anterior deltoid more than the pectoralis major [15].

**Figure 1:**
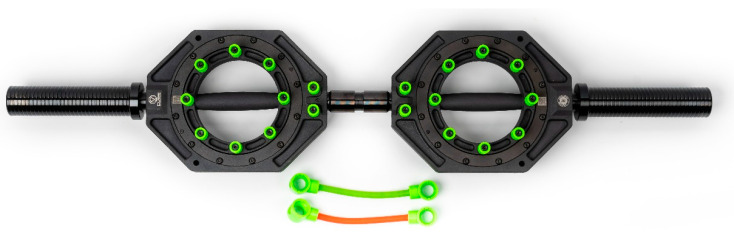
A model of the complete upper body bar (CUBB) device, where the handles are set at horizontal/neutral positions. The green band was used in this study (a courtesy picture from Resistance in Rotation Corporation, Peru, IN, USA).

Despite various bench press variations, traditional exercises do not target forearm pronation or supination, which led to the development of the complete upper body bar (CUBB) (Figure 1). The CUBB was designed to have the flexibility to add variable resistances to forearm pronation/supination in the transverse plane during standard lifts [16]. This novel CUBB device also has the simplicity of a traditional bar bell in allowing weights to be placed on both ends of the bar so adjustable resistance can be applied vertically in the sagittal plane. It seems feasible that when the CUBB is used during common upper body resistance exercises such as a bench press, forearm muscles can be included in training along with the primary muscles (shoulder muscles, upper arm muscles, etc.) typically targeted by the exercise. This could be significant because athletes of many sport events will have a convenient way to enhance their upper body strength profile via using the CUBB device. For example, competitive bodybuilders could realize the benefits of strengthening their forearm pronators and supinators during a regular bench press with a CUBB. Similarly, baseball players have a need to improve their forearm strength to enhance performance and prevent injuries along with their chest and shoulder muscles, which power the throwing motion [17]. With a CUBB, baseball players could address these upper body muscles effectively.

The efficacy of strengthening the upper body muscles via using a CUBB device during a regular upper body exercise such as a bench press has yet to be determined. Specifically, it was necessary to determine whether using a CUBB could have comparable effects on the shoulder and upper arm muscles to those associated with traditional devices during a bench press. Furthermore, it was important to know whether a CUBB device could effectively deliver resistance in the transverse plane to engage the forearm pronators and supinators. Therefore, the purpose of this study was to evaluate the neuromuscular activation profiles of the anterior deltoid (AD), triceps brachii (TRI), pectoralis major (PEC), pronator teres (PRO), and supinator (SUP) muscles during bench press exercises using a CUBB compared to traditional devices such as a bar bell (BB) or dumbbell (DB). We expected similar activation levels in the shoulder and upper arm muscles (AD, PEC, and TRI) with the CUBB compared to traditional devices (BB and DB). We also hypothesized that the forearm muscles (PRO and SUP) would be significantly more active when challenged by the CUBB compared to the two traditional devices.

## 2. Methods

Twenty-one healthy college-aged men (age: 21 ± 2 years; body mass: 88 ± 15 kg; body height: 181 ± 5 cm) volunteered for this study. Participants self-reported that they regularly participated in moderate-intensity resistance training three or more times per week based on American College of Sports Medicine (ACSM) guidelines [18]. Participants were free of a history of upper extremity surgery or injury that caused them to be contraindicated from resistance training for greater than six weeks. In addition, participants were free from any neurological, vestibular, or other disorder that affected their ability to safely perform upper body resistance training. This study was approved by the Ball State University Institutional Review Board (IRB protocol #1985725).

Upon arrival to the biomechanics laboratory on the day of testing, participants were provided a health history questionnaire and an informed consent document to fill out and sign prior to participation in the testing procedures. Participants were then provided with standard compression clothing and shoes. Anthropometric data were collected, including height, body mass, elbow width, wrist width, hip width, knee width, ankle width, leg length, and arm length. The skin was then prepped for placement of Delsys Trigno electromyography (EMG) sensors (Delsys Inc., Boston, MA, USA) by cleansing, shaving, and abrading the areas. EMG sensors were placed according to standard recommendations to collect activity from the AD, PEC, TRI, PRO, and SUP [19]. As all participants were right-hand dominant, and a pilot study confirmed similarities in muscle activations between both sides during each bench press condition, EMG data were collected from the right side of the body to reduce the size of the data file. Specifically, the AD EMG sensor was placed three fingerbreadths below the anterior margin of the acromion. The EMG sensor on the PEC was placed below the clavicle but above the nipple and breast, with electrodes parallel to the ground. The TRI EMG sensor was placed on the long head, four fingerbreadths distal to the posterior axillary fold. The PRO EMG sensor was placed two fingerbreadths distal to the midpoint of a line connecting the medial epicondyle and biceps tendon. The SUP EMG sensor was placed just radial to the most distal part of the insertion of the biceps tendon. EMG sensors were set to sample at 2000 Hz. EMG signals were band-pass-filtered (20–450 hz) during data acquisition.

Following EMG sensor placement, reflective markers were applied to the participant in a modified Plug-in Gait (Vicon Motion System Ltd., Oxford, UK) arrangement. Once participant preparation was complete, the participant practiced a few lifts with the different devices without any added weight in order to familiarize them with the protocols. Weights were then added to the devices to obtain 30% of the participant’s body weight (BW). A load of 30% of BW normally represents a level of light to moderate resistance for average American males. Using light-to-moderate resistance would reduce the likelihood of developing fatigue during the testing. The cadence of all exercises and conditions were regulated using a metronome set at 30 Hz [20]. Motion capture data were collected during trials using 12 Vicon Vantage and Vero Motion Capture cameras and Vicon Nexus v15 (Vicon Motion System Ltd., Oxford, UK), sampling at 100 Hz.

The bench press conditions included the BB, DB, and CUBB. The order of the conditions was randomly determined for each participant. During each bench press condition, four repetitions were performed. A five-minute break was given to the participant between conditions. General forms of bench press followed the International Powerlifting Federation’s recommendations [21]. The participant’s body including the back, head, shoulders, and buttocks were in contact with the bench surface; the feet were flat on the floor. All bench presses were performed using a standard grip style. The bench press movement began with taking the device with the upper limbs extended while lying on a bench. The device was then lowered to the chest for a momentary pause while the device was motionless; the device was then symmetrically pushed up to a position where the upper limbs were fully extended. Additionally, for the BB condition, the participant grasped the bar with an overhand grip just wider than shoulder width and held it above the chest with arms fully extended. For the CUBB condition, the device was configured to introduce a 30 inch-pound rotational resistance in the transverse plane via a calibrated rubber band. In the up position, the CUBB handles were held in a horizontal position. The CUBB device was then lowered to the middle chest while handles were rotated into a neutral grip position. During the lift, the handle grips were then slowly rotated to a horizontal position while the bar was pushed up and returned to its starting position. For the DB condition, in the up position, the participant held the device with elbows fully extended and forearms in a full pronation position. The DB device was then lowered to the down position where the elbow was at 90° angle and the forearm was in a semipronated position. During the lift, the participant pushed the DB devices up until the arms were fully extended and the forearms were pronated.

Following the completion of all condition trials, the participants completed maximum voluntary isometric contraction (MVIC) trials for each muscle group of interest in order to normalize the EMG data. Visual 3D v6 software (C-Motion Inc., Germantown, MD, USA) was used to create a biomechanical model and perform EMG data processing. The root mean square (RMS) procedure was used to condition the raw EMG data with a 100 ms moving window (V3D, C-Motion Inc., Germantown, MD, USA). Specifically, the following equation was used: RMS = {1/T∫_0_^T^ (rEMG(t))^2^dt}^1/2^, where rEMG(t) represents the raw EMG at time t, dt is the sampling rate (0.5 ms), and T is the moving window with a size of 100 ms. The individual muscle’s RMS EMG during movements were then normalized to the RMS EMG of their MVIC for data analysis purposes. The biomechanical model was then used to determine critical events (starting positions and ending positions) and phases (concentric and eccentric phases) of the bench press movements.

Repeated-measures analysis of variance (ANOVA) was performed in SPSS v22 (IBM, Armonk, NY, USA) to determine the differences in the muscle activation patterns between the CUBB and the DD and BD conditions. The independent variable was the bench press condition. The dependent variables were the normalized peak RMS EMG of the muscles during concentric actions. Significance level was at *p* ≤ 0.05. Furthermore, to complement the hypothetical analysis conducted in this study, so that a better understanding of the implication of the statistical inferences can be obtained, a set of relevant statistical parameters are also reported. These statistical measures include *p*-value and effect size. A *p*-value (ranging from 0 to 1) is a statistical measure of the likelihood of making an error in correctly explaining the statistical findings. A *p*-value less than the established threshold of 0.05 (alpha = 0.05) indicates that there is a less than 5% chance that the statistical inference is wrong. The effect size, represented by eta squared (µ^2^) (ranging from 0 to 1), is a statistical measure quantifying the strength of the effect of the independent condition on the dependent variables. Typically, µ^2^ values between 0.01 and 0.06, between 0.06 and 0.14, and over 0.14 are considered a small effect, a medium effect, and a large effect, respectively [22].

## 3. Results

Repeated-measures ANOVAs revealed significant differences in muscle activations during the three bench press conditions (*p* < 0.001). Table 1 shows the *p*-values and effect sizes of the ANOVA tests. Table 2 shows the means and standard deviations (SDs) of the peak RMS EMGs of the AD, PEC, TRI, PRO, and SUP during the concentric phase under the bench press conditions. For the shoulder and chest muscles (the AD and PEC), there were no significant differences in the peak RMS EMGs among the three conditions (*p* = 0.642 and *p* = 0.526, respectively). Both the main tests for the AD and PEC revealed large *p*-values (*p* > 0.05). In addition, the effect sizes of the AD and PEC tests were less than 0.06, which indicated small effects of the bench press condition on these muscles’ activation profiles. Thus, the lack of differences in magnitude seen in the PEC and AD across the conditions supported the notion that all three bench presses required a similar level of engagement of the PEC and AD.

For the upper arm muscle (the TRI), there was a significant main effect in the peak RMS EMG (*p* = 0.049). The small *p*-value (*p* < 0.05) of the main test combined with the large effect size (µ^2^ = 0.14) indicated that the bench press condition had a significantly large effect on the behavior of TRI activation. Specifically, the CUBB condition showed a greater peak RMS EMG of the TRI than the DB condition (23% greater, *p* = 0.024). The BB condition also showed a trend of a greater peak RMS EMG of the TRI than the DB condition (20% greater, *p* = 0.051).

For the forearm muscles (the PRO and SUP) tested, there was a significant main effect in PRO activation among the conditions (*p* = 0.045). The *p*-value of the main test was significantly smaller than the established threshold (alpha = 0.05). Also, there was a large effect size (µ^2^ > 0.14) detected in the test. The significantly small *p*-value combined with the large effect size (µ^2^ = 0.143) indicated that the bench press condition had a strong effect on the PRO’s activation level. Specifically, the peak RMS EMG of the PRO muscle was 41% higher with the CUBB than with the DB (*p* = 0.027). Also, the peak RMS EMG of the PRO muscle in the CUBB condition was 37% higher than that in the BB condition (*p* = 0.063). Furthermore, there was a significant main effect in the SUP activation among the conditions (*p* < 0.001). The *p*-value of the main test was significantly small (*p* = 0.001). In the meantime, there was a significantly large effect size (µ^2^ = 0.332) detected. The significantly small *p*-value combined with the large effect size indicated that the bench press condition had a strong effect on the SUP’s activation profile. Specifically, the peak RMS EMG of the SUP muscle with the CUBB was 67% and 30% higher than with the BB (*p* < 0.001) and DB (*p* = 0.006), respectively. In summary, it is evident that both the forearm muscles (PRO and SUP) were pronouncedly more engaged under the CUBB condition than under the other two conditions.

## 4. Discussion

The purpose of this study was to examine the activation profiles of the upper body muscles represented by the AD, PEC, TRI, PRO, and SUP during a variety of bench press exercises. Three bench press conditions were performed via using a CUBB, a BB, and a DB. It was hypothesized that the CUBB condition would require greater forearm muscle engagement than the other two standard bench press conditions. This hypothesis was largely supported by the results. It was found that the PRO muscle was significantly more active during the CUBB condition than the DB condition and showed a trend of a higher level of engagement than under the BB condition. We also hypothesized that the three main movers (AD, PEC, and TRI) in the bench press exercise would demonstrate comparable activation levels among the three conditions. This hypothesis was largely supported. All three bench press conditions showed similarly high levels of EMG activities in the AD and PEC muscles. However, the DB seemed to generate less TRI activation than the BB and CUBB.

The bench press is a continuous movement; multiple repetitions are performed during one set of exercise. In a typical bench press cycle, there is an eccentric phase and a concentric phase. The eccentric phase begins with the device held in an up position and finishes when the device is lowered to a down position. During this time, the major movers, such as the anterior deltoid, pectoralis major, triceps brachii, perform eccentric actions to control the downward movement of the device. The concentric phase sees a reversed action of the eccentric phase: it begins with the device held in a down position and finishes when the device is pushed to an up position. During this time, the major movers are acting concentrically to elevate the device to the up position.

During the concentric phase of a bench press, the shoulder goes through a range of flexion and adduction, while the elbow joint goes through an extension. The muscles responsible for these joint movements are activated at a high level and produce strong forces to help the body overcome resistance and move the training devices to an up position. All three exercise devices tested in this study were found to be very effective in delivering resistance (30% of BW weight) to the body. We found that no matter which device was used, all three major movers (PEC, AD, and TRI) exerted intense activities during the concentric phase of the press. Compared to the DB condition, the CUBB condition seemed to better resemble the outcomes of the BB condition, with all the major muscles demonstrating similar levels of activation between the two conditions. However, the DB condition showed a 20% lower activation of the TRI compared to the other two bar conditions. It is possible that due to the nature of the dumbbell device, each individual dumbbell must be managed independently during a bench press. To achieve a symmetrical form of exercise, there needs to be relatively high skill and coordination between the left and right upper extremities. To ensure the DB device is lifted to the targeted up position from a down position, the muscles of the distal joints along the open kinetic chain such as the elbow extensors might play the role of a facilitator or an assistant mover to help the other primary muscles from the chest and shoulders to complete the lift. Thus, a slight deviation in the TRI activation was present between the DB and the bar conditions. More studies are necessary to understand the role of the TRI during a bench press when different weight devices are used.

In addition to the basic shoulder and elbow movements, there was a forearm motion integrated into two of the three conditions. The CUBB condition required 90° pronation during the concentric phase. The DB condition also required 90° forearm pronation. Due to the constant resistance applied in the transverse plane (a 30 inch-pound torque), the CUBB device effectively challenged the pronators during the forearm pronation. In the meantime, the SUP, serving as an antagonist, elevated its co-contraction level to control the forearm movement in the pronation direction. Apparently, introducing a constant rotational resistance in the transverse plane helps to promote engagement of the PRO and SUP. The high yield of muscle force and EMG activity from the PRO and SUP are predictable outcomes when using the CUBB. In this study, the additional rotational resistance of a 30 inch-pound torque applied in the transverse plane allowed the CUBB device to place a high demand on more forearm muscle involvement. Specifically, the PRO with the CUBB was nearly 40% more challenged than under the DB and BB conditions, and the SUP with the CUBB worked at least 30% harder than those under the DB and BB conditions. However, a high activation level of the PRO and SUP was not evident under the DB condition. Theoretically, during a bench press, the dumbbell is a free weight attached to the end of an open kinetic chain (the hand); resistance from a dumbbell weight is effectively applied to the kinetic chain in the sagittal plane; however, in the transverse plane, the rotational resistance available for challenging the forearm muscles is low. It was evident that it did not take too much of an effort for the PRO to initiate forearm pronation during the press. In the meantime, the SUP’s co-contraction level was kept at a low level as well. The EMG data further showed that the pattern of the relatively low activation level of the PRO and SUP was quite similar between the standard BB condition and the DB condition.

Upper body muscular strength/endurance training is an essential element of a physical conditioning program. Common upper body exercises such as the bench press are designed to target a limited number of joints and muscles. This is largely attributed to the nature of the exercise devices used, which are limited to delivering resistance in one direction or within a single plane. In order to further condition other muscles along the upper body kinetic chain, separate training protocols must be implemented to address those muscles. For example, the traditional bench press protocol with a BB or a DB can only effectively address the shoulder and upper arm muscles (AD, PEC, and TRI); forearm muscles (PRO and SUP) must be trained separately through a different protocol such as a hammer rotation. The CUBB is designed to deliver adjustable resistances to the body in two planes (sagittal plane and transverse plane); thus, the major muscles along the kinetic chain of the upper body can be effectively targeted. For example, when executing a bench press maneuver with a CUBB, the body has to overcome resistance in the sagittal plane using the shoulder and upper arm muscles (AD, PEC, and TRI) and resistance in the transverse plane using the forearm muscles (PRO and SUP). The findings from this study indicate that the CUBB is effective in complementing a regular bench press procedure, with additional muscles being trained. Thus, using a CUBB during common upper body exercises such as a bench press is a convenient and effective way to achieve a complete training goal.

The outcomes of this study can be further applied in the clinical field for improving the quality of life of the patient population. For example, osteoporosis is a major health problem affecting hundreds of millions people worldwide [23]. This pathological condition is a bone disorder that degrades bone strength and leads to an increased risk of fracture. Middle-aged women experiencing postmenopausal changes face accelerated bone loss and develop osteoporosis [23]. Older adults inevitably live with osteoporotic conditions, with a noticeable decline in bone strength as a result of the aging process [24,25]. Low bone strength combined with a fall can result in devastating consequences such as bone fractures. In fact, wrist fracture is one of the most common fractures after a fall, and fall-related incidents account for 95% of wrist fractures [26]. Thus, it is a real challenge for healthcare professionals to find ways to mitigate the fracture risk associated with osteoporosis. The benefits of using a CUBB in the osteoporotic patient population are twofold. First, it helps strengthen forearm muscles. The forearm muscles including the PRO and SUP can be effectively trained to enhance their ability to alleviate impact forces during a fall. Second, it helps improve forearm bone strength. The ulna and radius can accelerate their remolding process to meet the demand for supporting forearm muscle development during exercise. This process will result in increases in bone mass and both strength. Thus, adding a CUBB protocol into an individual’s preventative exercise program will allow healthcare professionals to effectively address the risk of osteoporosis related wrist fractures and help their patients overcome the fear of fall-related injuries.

Ordinary people will also realize the benefits of exercises incorporating the CUBB. Prospective observational studies suggested that negative health outcomes are inversely related to regular physical activity [27,28]. Common diseases such as cardiovascular disease, thromboembolic stroke, hypertension, type 2 diabetes mellitus, osteoporosis, obesity, colon cancer, breast cancer, anxiety, and depression are linked to high levels of physical inactivity [27,28]. The World Health Organization (WHO) and ACSM have developed guidelines for promoting physical activity among healthy adults [18,27,29]. For example, the general population is recommended to work out at a moderate/intensive level for at least 150 min per week for health benefits [18,27,29]. This guideline for maintaining a level of 150 min of moderate-intensity aerobic exercise during a week aims to promote the health of the cardiorespiratory system [18,27,29]. In addition to cardiorespiratory health, it is also essential to have good musculoskeletal system health. The ACSM regularly advocates resistance training for health and encourages people of all ages to participate in resistance exercise to obtain more health benefits [30]. The benefits associated with muscle-strengthening activity are significant. By performing muscle-strengthening exercises, individuals could maintain their physical independence, increase lean muscle mass, improve balance, reduce the risk of falls, and prevent bone loss. The WHO has provided guidelines for conditioning the musculoskeletal system [27,29]. It is recommended that adults of all ages participate in weight lifting exercises on a regular basis at a minimum of twice a week [27,29]. It is desirable that all major muscles in the body should be conditioned. Given the unique design feature of the CUBB system, lifting with a CUBB could effectively address many major upper body muscles. With the potential for enhancing the quality of upper body resistance training, the CUBB device can be a means for ordinary people to use to improve their musculoskeletal condition. Thus, the goal of gaining health-related benefits by following the ACSM and WHO guidelines could be better achieved.

Furthermore, the findings of this study could also valuable for the athletic population who may consider use of the CUBB in their training. Athletes performing striking or throwing events could use the CUBB to train their upper body musculature more efficiently. Along with an upper body strength gain, individuals could improve the coordination between the joints and the synergy between muscles when using a CUBB to execute bench presses. This is critical when a great emphasis is placed on energy transfer within the upper body kinetic chain during a striking or a throwing event.

As many studies are associated with limitations, this study is no exception. The current study recruited a cohort that was a group of college-aged male students. Apparently, the results from this study are more representative of the responses from men in the same college age range. Data from men in other age groups such as middle-aged and older-aged individuals could be collected for comparison purposes. In addition, women counterparts could benefit from studies performed in their representative age groups as well. Given the fact that individuals could have various weight training experiences, the biomechanical responses could be different between a beginner and an experienced trainee. It is necessary to expand this study to investigate how weight training experience influences the muscles’ responses with a CUBB.

The current study focused on the acute effect of a bench press on muscle responses. It is unclear how muscles respond to the same exercise protocols after a typical training session. Future longitudinal studies are warranted to monitor the training effects on the changes in the muscles’ responses, muscle strength gain, and improvement of movement performance over multiple weeks and beyond. It is also desired to understand the effect of CUBB training over a longer term on the quality of the remodeling process in the wrist bones. This is significant given the urgency of developing preventive exercise programs to help combat osteoporosis. In addition to using EMG as a means to understand muscle responses, other biomechanical and physiological variables could be collected. These variables include cross-sectional areas of the target muscles, maximum strength of the upper body joints, forearm bone mass and density, and levels of perceived exertion during exercise. Also, athletes will benefit from knowing the gain in their athletic performance after using the CUBB through a regular training season. As artificial intelligence (AI) becomes a powerful tool to develop individualized training programs, the data collected in this study and from future studies could be used by AI to identify the optimal training frequency for individuals, to determine effects of different exercise intensities on muscle strength and movement performance, and to describe the synergistic effects obtained when other training exercises are combined with upper body training with a CUBB.

## Figures and Tables

**Table 1:** *p*-values and effect sizes of the repeated-measures analysis of variance (ANOVA) tests.

Muscles	Significance (*p*-Value)	Effect Size (Partial µ^2^)
AD	0.642	0.022
PEC	0.526	0.032
TRI	0.049	0.140
PRO	0.045	0.143
SUP	0.001	0.332

Note. AD: anterior deltoid; PEC: pectoralis major; TRI: triceps brachii; PRO: pronator teres; SUP: supinator.

**Table 2:** Means and standard deviations of peak RMS EMG (%MVIC) of AD, PEC, TRI, PRO, and SUP during the concentric phase of a bench press.

Muscles\Conditions	CUBB	BB	DB
AD	98 ± 76	101 ± 80	104 ± 66
PEC	101 ± 87	117 ± 114	102 ± 52
TRI	100 ± 81 ^#^	97 ± 71	81 ± 65 ^#^
PRO	100 ± 83 ^#^	73 ± 67	71 ± 65 ^#^
SUP	87 ± 70 * ^#^	52 ± 47 *	67 ± 68 ^#^

Note. CUBB: complete upper body bar; BB: bar bell; DB: dumbbell; AD: anterior deltoid; PEC: pectoralis major; TRI: triceps brachii; PRO: pronator teres; SUP: supinator. * Significantly different between CUBB and BB (*p* < 0.05); ^#^ significantly different between CUBB and DB (*p* < 0.05).

## Data Availability

The data presented in this study are available upon request from the corresponding author.

## References

[1] Jeon J.Y., Kim J.H., Kwon O.Y. (2023). The relationship between upper extremity, trunk and hip muscle strength and the modified upper quarter Y-balance test. Phys. Ther. Korea.

[2] Gonzalez-Galvez N., Gea-Garcia G.M., Marcos-Pardo P.J. (2019). Effects of exercise programs on kyphosis and lordosis angle: A systematic review and meta-analysis. PLoS ONE.

[3] Wang Z., Zan X., Li Y., Lu Y., Xia Y., Pan X. (2023). Comparative efficacy different resistance training protocols on bone mineral density in postmenopausal women: A systematic review and network meta-analysis. Front. Physiol..

[4] Kraemer W.J., Ratamess N.A. (2005). Hormaonal responses and adaptations to resistance exercise and training. Sports Med..

[5] Salinero J.J., Coso J.D. (2022). Rotational versus glide technique in elite shot put: Trend analysis in the 21st century. J. Hum. Sport. Exerc..

[6] Fortenbaugh D., Fleisig G.S., Andrews J.R. (2009). Baseball pitching biomechanics in relation to injury risk and performance. Sports Health.

[7] Serrien B., Baeyens J.P. (2018). Systematic review and meta-analysis on proximal-to-distal sequencing in team handball: Prospects for talent detection?. J. Hum. Kinet..

[8] Floyd R.T. (2023). Manual of Structural Kinesiology.

[9] Schick E.E., Coburn J.W., Brown L.E., Judelson D.A., Khamoui A.V., Tran T.T., Uribe B.P. (2010). A comparison of muscle activation between a Smith machine and free weight bench press. J. Strength Cond. Res..

[10] Saeterbakken A.H., Fimland M.S. (2013). Electromyographic activity and 6RM strength in bench press on stable and unstable surfaces. J. Strength Cond. Res..

[11] Stastny P., Gołaś A., Blazek D., Maszczyk A., Wilk M., Pietraszewski P., Petr M., Uhlir P., Zając A. (2017). A systematic review of surface electromyography analyses of the bench press movement task. PLoS ONE.

[12] Barnett C., Kippers V., Turner P. (1995). Effects of variations of the bench press exercise on the EMG activity of five shoulder muscles. J. Strength Cond. Res..

[13] Lehman G.J. (2005). The influence of grip width and forearm pronation/supination on upper-body myoelectric activity during the flat bench press. J. Strength Cond. Res..

[14] McCaw S.T., Friday J.J. (1994). A comparison of muscle activity between a free weight and machine bench press. J. Strength Cond. Res..

[15] Rodriguez-Ridao D., Antequera-Vique J., Martin-Fuentes I., Muyor J.M. (2020). Effect of five bench inclinations on the electromyographic activity of the pectoralis major, anterior deltoid, and triceps brachii during the bench press exercise. Int. J. Environ. Res. Public Health.

[16] https://resistanceinrotation.com.

[17] Fan S., Cepek J., Symonette C., Ross D., Chinchalkar S., Grant A. (2019). Variation of grip strength and wrist range of motion with forearm rotation in healthy young volunteers aged 23 to 30. J. Hand Microsurg..

[18] Riebe D. (2018). ACSM’s Guidelines for Exercise Testing and Prescription.

[19] Criswell E. (2011). Cram’s Introduction to Surface Electromyography.

[20] Calatayud J., Borreani S., Colado J.C., Martin F.F., Rogers M.E., Behm D.G., Andersen L.L. (2014). Muscle activation during Push-Ups with different suspension training systems. J. Sports Sci. Med..

[21] IPF (2024). Technical Rules Book of the International Powerlifting Federation.

[22] Cohen J. (1988). Statistical Power Analysis for the Behavioral Sciences.

[23] Lane N.E. (2006). Epidemiology, etiology, and diagnosis of osteoporosis. Am. J. Obstet. Gynecol..

[24] U.S. Department of Health and Human Services (2004). Bone Health and Osteoporosis: A Report of the Surgeon General.

[25] Seeman E. (2003). Physiology of aging. Invited review: Pathogenesis of osteoporosis. J. Appl. Physiol..

[26] Dontas I.A., Yiannakopoulos C.K. (2007). Risk factors and prevention of osteoporosis-related fractures. J. Musculoskelet. Neuronal Interact..

[27] Haskell W.L., Lee I.M., Pate R.R., Powell K.E., Blair S.N., Franklin B.A., Macera C.A., Heath G.W., Thompson P.D., Bauman A. (2007). Physical activity and public health: Updated recommendation for adults from the American College of Sports Medicine and the American Heart Association. Med. Sci. Sports Exerc..

[28] Kesaniemi Y.A., Danforth E., Jensen M.D., Kopelman P.G., Lefebvre P., Reeder B.A. (2001). Dose-response issues concerning physical activity and health: An evidence-based symposium. Med. Sci. Sports Exerc..

[29] Bull F.C., Al-Ansari S.S., Biddle S., Borodulin K., Buman M.P., Cardon G., Carty C., Chaput J.-P., Chastin S., Chou R. (2020). World Health Organization 2020 guidelines on physical activity and sedentary behavior. Br. J. Sports Med..

[30] Fiataraone Singh M., Hackett D., Schoenfeld B., Vincent H.K., Wescott W. (2019). ACSM Resistance Training for Health. https://chapters.acsm.org/docs/default-source/files-for-resource-library/resistance-training-for-health.pdf.

